# Self-managed digital technologies for pressure injury prevention in individuals with spinal cord injury: a systematic scoping review

**DOI:** 10.1038/s41393-025-01113-w

**Published:** 2025-08-18

**Authors:** Samuel David Williamson, Maria Zofia Geisler, Randi Kjær Steensgaard, Knærke Søgaard, Søren Steen Nielsen, Line Trine Dalsgaard, Sophie Lykkegaard Ravn

**Affiliations:** 1 Specialized Hospital for Polio and Accident Victims, Rødovre, Denmark; 2 Department of Psychology, University of Southern Denmark, Odense, Denmark; 3 Research Unit for Plastic Surgery, Odense University Hospital, Odense, Denmark; 4 Department of Clinical Research, University of Southern Denmark, Odense, Denmark; 5 Skin Integrity Research Group (SKINT), Ghent University, Ghent, Belgium

**Keywords:** Disease prevention, Skin manifestations

## Abstract

**Study design:**

Systematic scoping review.

**Objectives:**

Pressure injuries (PI) are a serious but mostly preventable complication associated with living with spinal cord injuries (SCI). This review aims to identify and summarize evidence concerning self-managed digital technologies for preventing PI in the SCI population.

**Methods:**

A systematic search was performed across seven databases—Embase, Medline, PsycInfo, Web of Science, Scopus, CENTRAL, and CINAHL. To be eligible, studies had to be peer-reviewed and report original findings on self-managed digital technologies for PI prevention in adults (≥18 years) with SCI. Supplementary searches were conducted using Google Scholar, PEDro, and citation tracking to locate relevant studies not identified by the systematic search. Data from the included studies were extracted and synthesized.

**Results:**

The systematic search identified 9797 unique studies. After screening and excluding 8939 records at the title-and-abstract level, 858 full-text records were assessed, and 12 met the inclusion criteria. The included studies fell into categories: (i) technology-driven feedback systems that provide real-time pressure distribution data and (ii) digital self-management and educational systems aimed at improving adherence to PI-preventive measures. Feedback systems were associated with improved pressure-relieving behaviours, though adherence to reminder-based interventions remained a challenge. Digital self-management tools were shown to enhance knowledge and confidence related to PI prevention.

**Conclusions:**

Self-managed digital technologies increased awareness, confidence, and engagement in pressure relief behaviours among individuals with SCI. However, their direct impact on PI prevention remains inconclusive. Difficulties relating to adherence indicate that such technologies should complement, rather than replace, traditional prevention strategies.

## Introduction

According to recent international guidelines, pressure injuries (PI) are defined as “localized damage to the skin and/or underlying tissue, usually over a bony prominences or related to a medical or other devices, resulting from prolonged pressure or pressure in combination with shear” [1]. The complication is reported to impact approximately 12.8% of hospitalized adults worldwide [2]. In spinal cord injured (SCI) populations, the global pooled magnitude is significantly higher, estimated at around 32.36% [3]. This increased risk of developing a PI may be attributed to a series of changes in the skin and tissue experienced following a spinal cord injury [4], in addition to factors such as reduced mobility, increased spasticity, and poor skin sensitivity among individuals with SCI [5]. PIs are a serious complication associated with significant physical and psychological health problems. Affected individuals are at increased risk of developing sepsis and infection of the underlying bone or joint following PI [6], which may lead to further morbidity and pain [7]. Although bedrest is frequently recommended as part of PI management, prolonged periods of immobility can significantly limit participation in daily and community activities, thereby impacting overall quality of life [8, 9]. Moreover, severe PIs may be distressing due to their unpleasant appearance and odour, negatively influencing self-esteem and body image [10–12]. PIs have also been linked to more frequent and longer hospitalisations [13], higher treatment costs [14], and a reduced life expectancy in SCI populations [15]. Thus, prevention of PIs is an important matter both from a person-centered and a societal perspective.

Since most PIs are preventable [16], there is a strong rationale for prioritizing prevention and early detection over treatment. Effective preventive strategies include regular skin assessments, optimization of nutritional status and hydration, use of appropriate support surfaces, and consistent repositioning practices [17]. In parallel with these recommendations, recent studies highlight an emerging role for digital technologies in PI prevention among individuals with spinal cord injury (SCI). These innovations include tools for continuous pressure monitoring and other smart devices, many of which are designed to support self-management during rehabilitation (i.e., independent monitoring, prevention, and response) by enhancing autonomy and patient education [18–20]. Despite increasing interest in digital, user-empowering solutions to improve healthcare delivery, no comprehensive attempt has yet been made to identify, evaluate, and synthesize empirical evidence on self-managed digital technologies for PI prevention in SCI rehabilitation. This systematic scoping review addresses this critical knowledge gap to inform future research and clinical practice.

## Methods

The Preferred Reporting Items for Systematic Reviews and Meta-Analyses Extension for Scoping Reviews (PRISMA-ScR) [21] provided the framework for the overall production of this systematic scoping review. The protocol was registered in Open Science Framework on 12th July 2024 (10.17605/OSF.IO/TAE9C). The search strategy is reported according to the PRISMA-S [22].

### Search strategy

Two search blocks assembling search terms synonymous or closely associated with (a) PI and (b) SCI were developed. These search blocks were constructed based on a thorough reading of relevant literature and the authors’ prior subject knowledge. On 12th July 2024, the search blocks were run through seven bibliometric databases, chosen to cover a range of scientific disciplines. These databases were EMBASE (via OVID), PsycINFO (via OVID), Medline (via OVID), Scopus, Web of Science, CINAHL (via EBSCOhost), and CENTRAL, all searched from inception to 12th July 2024. The same search terms were entered across all databases at the title, abstract, and keyword level (ti,ab,kw) or similar. The search string was adapted to account for syntactic differences and variations in subject headings across indexed databases. Boolean operators were used to search both within (‘OR’) and between (‘AND’) blocks. No database restrictions were imposed, as all research designs from any given year were permissible per the eligibility criteria, described below. The full search strategy is available as Supplement 1 in the Supplementary Materials.

#### Additional search strategy

Records published in an ineligible format but considered thematically relevant were tagged and documented for additional searching. In addition to all included studies, tagged studies were subject to both forward snowballing [23] performed in Google Scholar [24]) and backward snowballing [23] (performed manually) on 27th November 2024. Five unique combinations of search terms from across the two search blocks (e.g., spinal cord lesion* + decubit*) were entered into Google Scholar on 13th November 2024. The first 50 results of each search sorted by both relevance and date were screened according to the eligibility criteria. Further, in PEDro [25], all clinical trials retrieved via a search for “spinal cord injury” were screened for potential relevance on 13th November 2024. If not already captured by the database searches, studies found via additional searches were imported into the online systematic review management tool Covidence [26] for independent screening at the full-text level by two reviewers. Additionally, the titles of all included studies were searched in Google to ensure no post-hoc retractions or corrections had been issued.

### Eligibility criteria

Included studies were required to meet all predefined eligibility criteria. Studies had to be published in a peer-reviewed journal in either English, Danish, Norwegian, or Swedish and include original data from human participants aged ≥18 years living with SCI. Additionally, studies had to have clinically evaluated the effect of any self-managed digital technology on preventing PI development. Reviews, book chapters, dissertations, editorials, letters, registrations, protocols, conference papers, and non-research articles were not included. Studies were excluded where SCI participants represented a minority percentage of the total study sample, or where subgroup results were not provided.

### Screening procedure

Records exported from the seven databases were imported into Covidence [26]. Duplicates were automatically detected, with half manually checked for accuracy by a trained student assistant. In groups of two, three authors and one student assistant independently pilot-screened the first 100 records at the title-and-abstract screening level to ensure proper and consistent use of the eligibility criteria. Internal screening guides were produced to assist the entire screening process. Further screening and discussion continued among four authors and one student assistant until a unanimous decision was reached on each record, with disagreements resolved internally. At the title and abstract level, uncertainties regarding the population, format, or intervention (e.g., whether an intervention could be considered digital, capable of optimization using digital technology, or was self-administered) were conservatively forwarded to check at the full-text level, providing the record was assumed to meet all other criteria. Where abstracts were missing, reviewers attempted to locate them online. Where an abstract was missing, but it was possible to confirm that the article was published in the wrong language or format, or where the title gave clear indication of irrelevance, the study was excluded. In all other circumstances, titles with no identifiable abstract were screened in a deliberately inclusive manner based on this information alone.

Forwarded materials were independently double-screened among four authors and a student assistant at the full-text level to determine the studies ultimately eligible for inclusion. At the full-text level, relevant interventions were forwarded irrespective of their administration in a clinical or a community setting, providing the intervention was self-administered, and the study otherwise met all other criteria. Reasons for exclusion were registered at the full-text level. Corresponding authors were contacted to provide additional information where ambiguity relevant to the decision-making progress arose. As an addendum to our a priori eligibility criteria, it was agreed during the screening process that case studies would be excluded on account of their low evidence quality [27, 28].

### Data extraction

The first author and a trained student assistant independently extracted data from the included studies. This data consisted of the author group, year of publication, country of production, study design, setting, sample size, sample characteristics (gender and age variables), injury characteristics (completeness, neurological level of injury, and time since injury), study outcome(s), intervention, and main results. Clarifications from corresponding authors were to be included in a final column marked ‘other’. Any discrepancies among the extractions were checked and discussed to reach a collective extraction table, also included for publication (Supplements 2 and 3).

### Synthesis methods

The screening process was summarized in the form of a flow diagram compliant with the PRISMA Guidelines [29]. The first author used the double-extracted data to perform a descriptive and narrative synthesis of the included studies. In the absence of a formal risk of bias assessment, study quality is addressed in the discussion section. The narrative synthesis consisted of drawing comparisons and disparities between studies to form a full overview of the current evidence across studies. These findings were organized into chapters that form the main body of the analysis, presented in the result section below.

## Results

### Study flow

**Fig. 1:**
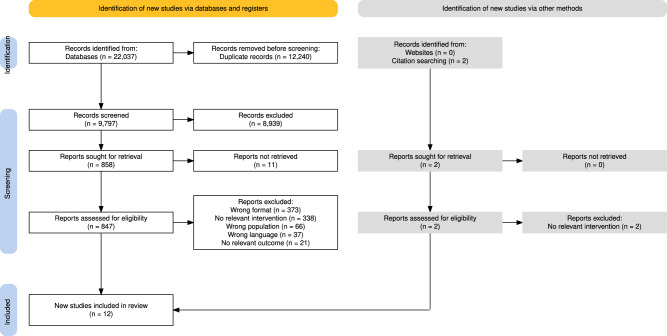
Flow diagram illustrating the stepwise study selection process.

A total of 9797 unique records were identified from the database searches. Following the exclusion of 8939 records at the title-and-abstract screening level, 858 full-text records were assessed for eligibility. At this stage, studies were excluded on account of their format (*n* = 373), intervention (*n* = 338), population (*n* = 66), language (*n* = 37), and outcome (*n* = 21). A further 11 studies were inaccessible (see Supplement 4). No relevant additional studies were identified through snowballing or unsystematic searches. In summation, 12 studies met the inclusion criteria for this review. Figure 1 visualises the study flow.

### Study characteristics

Among the 12 included studies, four were randomized controlled trials, four were pre-post intervention studies, two were feasibility or pilot studies, one was observational, and one was qualitative. All studies were published between 2010 and 2024. Half of the studies (*n* = 6) were conducted in the USA, while the remainder were conducted across Europe (*n* = 3), East Asia (*n* = 1), North America (*n* = 1), and one via international collaboration. The sample sizes ranged from 4–142 participants, with men (*n* = 146) outnumbering women (*n* = 56). The mean age of participants ranged from 36.1–59.9 years. Injury characteristics were reported inconsistently and scarcely, limiting the ability to draw cross-sample comparisons. Fewer than half of the studies (*n* = 5) reported complete information on the level of injury for intervention participants, across which thoracic (*n* = 53), cervical (*n* = 34), and lumbar (*n* = 3) SCI were represented. Eight studies reported time since injury, with one early intervention provided to participants an average of 3.7 months post-injury, while the remaining seven studies reported post-injury durations ranging from 5.8–19.4 years.

### Narrative synthesis

Findings were grouped according to intervention type and were organized under two core themes: (i) Technology-driven feedback systems providing real-time data of pressure distribution (*n* = 6) and (ii) Digital self-management and educational systems aimed at enhancing knowledge and adherence to preventative measures (*n* = 6).

#### Technology-driven feedback systems

Six studies leveraged digital technologies to monitor, record, and transmit real-time data to inform the pressure-relief activities of SCI participants [30–35]. The technology through which this information was delivered varied across the studies and was either (i) visual or (ii) tactile and/or auditory.

##### Visual feedback systems

Four studies utilized smartphone applications to provide real-time pressure mapping from wheelchair seat sensors [30–33]. The visual components of these applications varied, consisting of either colour-coded pressure maps indicating areas of low and high pressure, or visual cues related to posture, weight shifting, or reminders. In one study, nine SCI participants were monitored over three weeks: baseline (week 1), feedback via a smartphone app (week 2), and follow-up (week 3) [30]. Moderate effect sizes were observed between baseline and feedback (r = –0.628) and feedback and follow-up (r = 0.628), while the baseline–follow-up effect was small (r = 0.154). Feedback significantly improved the frequency (*p* = 0.001) and duration (*p* = 0.039) of pressure reliefs, though behaviours partially regressed after feedback was removed [30]. In another study, the introduction of interface pressure mapping increased participants’ confidence in performing pressure reliefs every 30 min, in holding pressure reliefs for 2 min, and in the belief that weight shifts prevent pressure injuries, although statistical significance was not reached (*p* > .05) [32]. During a four-week in-home trial of the Manual Wheelchair Virtual Coach, a daily average of only 2 lateral and 1.9 forward pressure-relieving leans across all participants was completed, while pressure-relieving attempts outnumbered completed actions, suggesting that participants were largely unsuccessful in maintaining postural changes despite receiving feedback [31]. However, the study reported no quantitative baseline data, which precludes any assessment of potential pre-post changes. Low compliance with pressure-relief reminders was also observed in a separate study [33]. While 39 (58.2%) of the 67 total local pressure alerts sent via the AW-Shift© app were acknowledged by participants, only 39 (11.2%) of the 349 total reminders were completed, with 306 (87.7%) reminders ignored completely [33].

##### Tactile & auditory feedback systems

Two studies communicated real-time pressure mapping from seat-mounted wheelchair sensors to participants via tactile or auditory feedback [34, 35]. One randomized controlled trial delivered electrostimulation to the tongue of four participants every 60 s to prompt and indicate the direction of required weight-shifting manoeuvres [34]. In comparison to a control group provided with no feedback, users exhibited greater improvements in adequate weight shifting and reported a lower rate of prolonged excessive pressure [34]. A quasi-experimental study reported similarly positive results from a one-week long investigation of pressure-relieving activities guided by an audio alarm system [35]. Compared to a week with no feedback, participants demonstrated statistically significant differences in four variables: fewer average daily minutes of uninterrupted sitting (84.36 ± 61.63 vs. 97.39 ± 73.68; *p* = 0.02), greater daily frequency of push-ups (3.35 ± 1.68 vs. 4.76 ± 3.33; *p* = 0.03), greater daily frequency of side-to-side leaning (4.25 ± 3.58 vs. 5.48 ± 5.62; *p* = 0.04), and a greater daily total frequency of pressure-relief activities (9.48 ± 6.35 vs. 12.30 ± 9.76; *p* = 0.01) [35].

#### Digital self-management & educational systems

Six studies evaluated digital self-management tools targeting PI prevention through educational modules, automated prompts, and interactive coaching [36–41]. These studies were categorized according to their level of engagement – either interactive or passive.

##### Interactive systems

Four digital self-management and educational systems allowed for bidirectional engagement, meaning participants could log and/or respond to programme content [36–39]. One study evaluated an internet-based skin care intervention that used branching logic to tailor the continual assignment of educational modules on topics such as pressure relief and nutrition [36]. After four weeks, all participants reported feeling better prepared to prevent PIs and to extend pressure relief duration. Additionally, 71% of participants found the programme beneficial for improving the frequency and regularity of their pressure-relieving activities [36]. A six-month analysis of CareCall, an interactive voice-response system for health monitoring, showed a significant resolution of PIs among all four women who reported a PI at baseline (*p* = 0.0001) [37]. The intervention demonstrated no significant effect on PI prevalence among men, nor an overall reduction in PIs at six months when controlling for baseline prevalence, age, and gender [37]. The iMHere educational app, which allowed participants to set pressure-relief reminders and record educational content, reduced the average frequency of wounds from 0.3, or approximately 6 wounds, to 0.1 per participant, or approximately 2 wounds, over nine months. However, this difference was not statistically significant (no *p*-value reported) [38]. An evaluation of the Pressure Ulcer Prevention and Management E-Learning Program, featuring an interactive case study, showed how 71.4% of participants felt ‘very sure’ that the programme improved confidence in preventing and detecting PIs, and ‘somewhat sure’ that they could enact programme recommendations [39]. Statistically significant improvements in skin and posture management (*p* = <0.05), as measured by questionnaire data, were also recorded [39].

##### Passive and standardized systems

Two studies offered unidirectional engagement with digital self-management and educational systems, where participants could not interact with the system or receive personalized feedback [40, 41]. In a 6-month pilot study, an automated SMS reminder system provided actionable tips for PI prevention, followed by postural and skin check reminders after four weeks [40]. By study completion, participants registered significantly greater adherence to pressure-relieving exercises (86.7% vs. 71.7%; *p* = 0.02) and reported being observant of PIs more days per week (6.75 vs 4.75, *p* = 0.04) compared to baseline [40]. On a 5-point skin care questionnaire, participants indicated feeling less inhibited from performing pressure relief (2.55 vs 2.73; *p* = 0.001) and less worried about the negative aspects of pressure relief (2.72 vs. 2.60; *p* = 0.04) at six months [40]. Participants also took the threat of PIs more seriously (67.1% at baseline vs. 69.6% at six months, *p* = 0.05) [40]. Of the 20 participants in the intervention group, four completed a satisfaction questionnaire, and all agreed or strongly agreed that the SMS system improved their confidence in PI prevention [40]. In another study, the staging and tissue healing modules of a custom educational app improved both participants’ knowledge and management of PIs [41]. One participant found that the app simplified prevention, while another reported that the app functioned as an effective reminder to perform weight-shift manoeuvres [41].

## Discussion

Several reviews have focused on behavioural and educational approaches to prevent PIs in individuals with SCI [42–45]. Others have examined compensatory or assistive technologies without the requirement that they be self-managed [46] or digital in format [18, 47, 48]. As such, the current review - exploring self-managed, digital technologies for preventing PI development in individuals living with SCI - addresses an important niche within the current scientific literature.

In total, 12 studies met the eligibility criteria [30–41]. Six studies relayed real-time pressure sensor data to participants via digital technology [30–35], while the remaining six studies utilized apps and e-learning platforms to deliver educational content designed to improve skin care and pressure-relief practices [36–41]. In summary, one internet intervention [36] and two feedback systems [30, 35] were reported to either improve pressure-relief frequency [30, 35, 36] and/or duration [30, 36] directly, or to improve confidence in performing these actions. In contrast, one study found that additional digital feedback failed to significantly improve confidence in either measure [32]. Two feedback systems were found to reduce the occurrence of prolonged pressure during sitting periods [34, 35]. Conversely, only non-significant [38] and gender-dependent [37] improvements in PI reduction were observed in two additional longitudinal mobile health interventions. Educational programmes were otherwise shown to increase knowledge, self-management, and PI monitoring practices [39, 41]. While one reminder system prompted improved adherence to pressure-relieving actions [40], another demonstrated the opposite [33]. Similarly, in one intervention using pressure sensor technology, participants recorded a higher percentage of attempted than completed pressure-relieving actions [31]. While it should be acknowledged that feedback systems inherently educate participants, suggesting a degree of conceptual overlap, the above synthesis organises studies by predominant intervention features and focus.

These findings suggest that the effectiveness of self-managed digital technologies in preventing PI development remains inconsistent. Comparable reviews report similar inconsistencies. For example, numerous reviews concerning behavioural and educational interventions [42], repositioning strategies [43], or general therapeutic interventions [44, 49], have found the evidence to be inconclusive or of low-quality.

Where reported, confidence [32, 39], knowledge [41], and preparedness [36] to manage PIs were consistently improved by the self-managed digital technologies examined in this review. However, in one educational intervention study, participants felt less confident in enacting programme recommendations than in detecting or preventing PIs [39]. Similarly, some studies reported challenges with translating knowledge into action [31] and in maintaining adherence [33, 35] to pressure-relief strategies supported by self-managed digital technologies, issues frequently raised in the broader PI prevention literature. For instance, Engelen et al. [50] questioned the legitimacy of equating improvements in knowledge-related outcomes alone, with overall improvements in self-management. Furthermore, O’Connor et al. [51] reviewed educational interventions for PI prevention in at-risk populations and found insufficient evidence to conclude that educational interventions reliably prevent PI development. These findings suggest that education alone may be insufficient to drive impactful behavioural change concerning PI prevention.

### Methodological considerations and limitations

Several qualifying factors went into our definition of a self-managed, digital technology. Numerous innovations presumed capable of self-management were excluded from the present review because they were still in the design phase [52, 53], were tested in case studies [54], or were not self-managed by the participants according to the study’s methods. One notable example of the latter comes from Oh et al. [55], whose chat-based mobile app, designed to facilitate PI prevention was, unbeknownst to the participants, dependent on human intervention rather than automated responses. This alludes to the broader point that interventions were no longer considered self-managed in context of the current study when clinicians directly engaged with participants, whether online or in person, to inform their pressure relieving behaviours. For instance, one study was excluded because a readout from a digital questionnaire was used to guide in-person consultations [56].

Our definition of digital technology is equally nuanced. Per the protocol, any analogue technology (e.g., a wheelchair cushion) optimized by digital technology (e.g., force sensors connected to a smartphone app) were eligible for inclusion, assuming other criteria were met [35]. During the extraction process, it was agreed that electrical stimulation would not be considered a digital technology unless it was digitally optimised or used for biofeedback purposes [57–63]. Studies evaluating digital technologies and analogue components together, where outcomes could not be attributed specifically to the digital technology, were also excluded [64].

Since no specific outcomes were pre-determined, included studies measured PI prevention in a variety of ways. As reported, this approach resulted in the inclusion of direct outcomes, such as PI reduction [37, 38], frequency of pressure-reliving behaviours [30, 31, 35, 36, 40], and duration of pressure exposure or relief [30, 31, 34, 36], and indirect outcomes, such as adherence to weight shift reminders [33], pressure relief knowledge acquisition [36, 39–41], and confidence levels in performing adequate pressure relieving behaviours [32, 39, 40]. Outcomes limited solely to the accuracy [65] or usability [66–70] of the technology of technology were excluded. It is worth noting that digital literacy was not reported in any included study. Without this information, it remains possible that low adherence or negative results may reflect poor user competence rather than the limitations of the technology itself.

As previously stated, it is acknowledged that pressure-relieving behaviours and how they are reported can vary considerably across studies. For example, one study [31] concedes that their technology only registered leans, which did not account for alternative pressure-relieving actions evidenced elsewhere, such as tilting [33] or push-ups [30, 32, 35]. This may explain why the positive quotes associated with app usage did not correspond with the quantitative data reflecting insufficient pressure-relief performance [31]. The results of included studies that measured only a small number of pressure relief outcomes should therefore be interpreted with this in mind.

### Avenues for future research and clinical implications

Based on the findings, there is an observed need for more robust study designs to evaluate the effectiveness of self-managed digital technologies in preventing PI development in SCI populations. While case studies were excluded during the screening process based on their low evidence quality, six of the included studies still reported sample sizes of under ten participants. Equally, statistical significance was either not reported or not achieved in studies. This limits the ability to draw meaningful conclusions about the collected evidence base, more so than the findings of individual studies. As observed by comparable reviews, more high-quality randomized controlled trials with larger and more diverse sample sizes are required to establish causality and strengthen the evidence-base [44, 49, 51].

While some studies tested their interventions in clinical settings [30, 34], a defining characteristic of self-managed technologies should be their usability in home and community settings. However, the present review also highlights challenges related to compliance when pressure-relief reminders are given in real-world settings [33]. Only four studies described participants receiving structured training prior to independent use of a digital technology [31, 32, 35, 41], suggesting researchers’ assumption of intuitive use. To increase user engagement, future research investigating the efficacy, rather than usability of digital technologies for PI prevention in SCI may consider offering pre-intervention training. Although this review focused exclusively on digital interventions, our search identified several electrical stimulation studies designed to assist SCI participants in PI prevention [57–63]. Many such examples can be self-administered or integrated into wearable technologies and therefore serve a similar purpose of increasing user agency and digital empowerment. A separate review assembling self-managed electrical stimulation interventions for pressure relief in SCI individuals could serve as a companion piece to the present article. This review primarily includes studies involving ‘digital immigrants’ [71] with mean ages ranging from 36.1–59.9, the increasingly ubiquitous nature of digital technology, both in healthcare and beyond, suggests that digital literacy across all age groups will rise in tandem in with technological advancements. As such, a future update to this review may be better positioned to determine the extent to which adherence is linked to digital literacy. Another possible explanation for poor adherence is presented in a recent qualitative study found that education provided in the early, inpatient phase of SCI rehabilitation was not meaningfully retained by participants [72]. Therefore, the delivery of any self-managed, digital intervention should be cognizant of participant readiness to receive information, both in hopes of targeting engagement, and out of respect for the physical and emotional adjustment to recently-acquired SCIs. Lastly, while findings are presented from educational interventions delivered via self-managed digital technologies, the present review cannot assess the wider evidence concerning non-digital educational techniques aimed at PI prevention for individuals with SCI. However, future research in health education might choose to compare different educational approaches to self-care practices in SCI groups.

As explained above, heterogeneity in injury characteristics and their reporting standards precluded a formal subanalysis of intervention effects moderated by relevant factors such as neurological level of injury, injury completeness, and time since injury. For example, one might assume that individuals with higher-level (e.g., cervical) SCIs may experience more significant limitations in performing pressure-relief manoeuvres than those sustaining lower-level (e.g., thoracic) SCIs, and that individuals with recently acquired injuries may benefit more greatly from educational content than those with long-established routines. While such assumptions are not empirically supported in the present review, they highlight important avenues for future research and should be considered when developing and testing future person-centred, self-managed digital technologies.

From a clinical perspective, issues related to adherence suggest that self-managed digital technologies should not be prescribed as standalone interventions for the prevention of PI development in SCI populations. However, while the statistical significance of individual results varied, a number of positive effects on both subjective and objective measures of pressure relief were observed, suggesting clinical relevance. As such, while pressure injury prevention remains elusive, these interventions may remain effective adjuncts to primary treatment when supplemented by clinical follow-up or behavioural reinforcement strategies to enhance engagement.

## Supplementary information

Supplement 1.

Supplement 2.

Supplement 3.

Supplement 4.

## References

[1] Kottner J, Worsley P, Brienza D. Pressure ulcers/injuries: definition and etiology. In: Prevention and treatment of pressure ulcers/injuries: clinical practice guideline. The international guideline. 4th Ed. 2025. https://www.internationalguideline.com/. Accessed 14 Aug 2025.

[2] Li Z, Lin F, Thalib L, Chaboyer W. Global prevalence and incidence of pressure injuries in hospitalised adult patients: a systematic review and meta-analysis. Int J Nurs Stud. 2020;105:103546. 32113142 10.1016/j.ijnurstu.2020.103546

[3] Shiferaw WS, Akalu TY, Mulugeta H, Aynalem YA. The global burden of pressure ulcers among patients with spinal cord injury: a systematic review and meta-analysis. BMC Musculoskelet Disord. 2020;21:334. 32471497 10.1186/s12891-020-03369-0 PMC7260823

[4] Gefen A. Tissue changes in patients following spinal cord injury and implications for wheelchair cushions and tissue loading: a literature review. Ostomy Wound Manage. 2014;60:34–45. 24515983

[5] Vecin NM, Gater DR. Pressure injuries and management after spinal cord injury. J Pers Med. 2022;12:1130. 35887627 10.3390/jpm12071130 PMC9325194

[6] Dana AN, Bauman WA. Bacteriology of pressure ulcers in individuals with spinal cord injury: What we know and what we should know. J Spinal Cord Med. 2015;38:147–60. 25130374 10.1179/2045772314Y.0000000234 PMC4397196

[7] Lyons S, Sorenson M. Quality of life in spinal cord injury patients with pressure ulcers. SCI Nurs. 2009;26:13–8.

[8] Lala D, Dumont FS, Leblond J, Houghton PE, Noreau L. Impact of pressure ulcers on individuals living with a spinal cord injury. Arch Phys Med Rehabil. 2014;95:2312–9. 25168376 10.1016/j.apmr.2014.08.003

[9] Soegaard K, Sig JR, Nielsen C, Verhaeghe S, Beeckman D, Biering-Sørensen F, et al. “I am just trying to live a life!” –a qualitative study of the lived experience of pressure ulcers in people with spinal cord injuries. J Tissue Viability. 2024;33:50–9. 38044163 10.1016/j.jtv.2023.11.009

[10] Harding-Okimoto MB. Pressure ulcers, self-concept and body image in spinal cord injury patients. SCI Nurs. 1997;14:111–7. 9510832

[11] Langemo DK, Melland H, Hanson D, Olson B, Hunter S. The lived experience of having a pressure ulcer: a qualitative analysis. Adv Skin Wound Care. 2000;13:225–35. 11075022

[12] Roussou E, Fasoi G, Stavropoulou A, Kelesi M, Vasilopoulos G, Gerogianni G, et al. Quality of life of patients with pressure ulcers: a systematic review. Med Pharm Rep. 2023;96:123–30. 37197280 10.15386/mpr-2531 PMC10184534

[13] Graves N, Birrell F, Whitby M. Effect of pressure ulcers on length of hospital stay. Infect Control Hosp Epidemiol. 2005;26:293–7. 15796283 10.1086/502542

[14] Tai C, Wang J, Lo S, Tsay S, Yang C, O Yang AC, et al. Incidence, prevalence, and medical costs of pressure injuries in Taiwan from 2001 to 2015: results of a retrospective cohort study. J Clin Nurs. 2024;34:1264–76. 38629347 10.1111/jocn.17149

[15] Krause JS, Saunders LL. Health, secondary conditions, and life expectancy after spinal cord injury. Arch Phys Med Rehabil. 2011;92:1770–5. 22032212 10.1016/j.apmr.2011.05.024 PMC3385509

[16] Wood J, Brown B, Bartley A, Margarida Batista Custódio Cavaco A, Roberts AP, Santon K, et al. Reducing pressure ulcers across multiple care settings using a collaborative approach. BMJ Open Qual. 2019;8:e000409. 31523723 10.1136/bmjoq-2018-000409 PMC6711432

[17] European Pressure Ulcer Advisory Panel, National Pressure Injury Advisory Panel and Pan Pacific Pressure Injury Alliance. Interventions for Prevention and Treatment of Pressure Injuries. In: Prevention and treatment of pressure ulcers/injuries: clinical practice guideline. The international guideline. 3rd Ed. https://www.internationalguideline.com/2019. Accessed 14 Aug 2025.

[18] Tung JY, Stead B, Mann W, Pham B, Popovic MR. Assistive technologies for self-managed pressure ulcer prevention in spinal cord injury: a scoping review. J Rehabil Res Dev. 2015;52:131–46. 26237111 10.1682/JRRD.2014.02.0064

[19] Fryer S, Caggiari S, Major D, Bader DL, Worsley PR. Continuous pressure monitoring of inpatient spinal cord injured patients: implications for pressure ulcer development. Spinal Cord. 2023;61:111–8. 35978113 10.1038/s41393-022-00841-7 PMC9970870

[20] Padula CA, Osborne E, Williams J. Prevention and early detection of pressure ulcers in hospitalized patients. J Wound Ostomy Continence Nurs. 2008;35:66–75. 10.1097/01.WON.0000308620.78884.88 18199940

[21] Tricco AC, Lillie E, Zarin W, O’Brien KK, Colquhoun H, Levac D, et al. PRISMA extension for scoping reviews (PRISMA-ScR): checklist and explanation. Ann Intern Med. 2018;169:467–73. 30178033 10.7326/M18-0850

[22] Rethlefsen ML, Kirtley S, Waffenschmidt S, Ayala AP, Moher D, Page MJ, et al. PRISMA-S: an extension to the PRISMA statement for reporting literature searches in systematic reviews. Syst Rev. 2021;10:39. 33499930 10.1186/s13643-020-01542-z PMC7839230

[23] Wohlin C. Guidelines for snowballing in systematic literature studies and a replication in software engineering. In: Shepperd MJ, Hall T, Myrtveit I (eds). Proceedings of the 18th international conference on evaluation and assessment in software engineering. Association for Computing Machinery (ACM), New York, 2014. pp 1–10.

[24] Google Scholar. Google Scholar. 2025. https://scholar.google.com.

[25] Physiotherapy Evidence Database (PEDro). PEDro - the Physiotherapy Evidence Database. 2025. https://pedro.org.au.

[26] Covidence systematic review software. Melbourne, Australia: Veritas Health Innovation. 2025. https://www.covidence.org.

[27] Murad MH, Asi N, Alsawas M, Alahdab F. New evidence pyramid. Evid Based Med. 2016;21:125–7. 27339128 10.1136/ebmed-2016-110401 PMC4975798

[28] Evans D. Hierarchy of evidence: a framework for ranking evidence evaluating healthcare interventions. J Clin Nurs. 2003;12:77–84. 12519253 10.1046/j.1365-2702.2003.00662.x

[29] Haddaway NR, Page MJ, Pritchard CC, McGuinness LA. PRISMA2020: an R package and Shiny app for producing PRISMA 2020‐compliant flow diagrams, with interactivity for optimised digital transparency and open synthesis. Campbell Syst Rev. 2022;18:e1230. 36911350 10.1002/cl2.1230 PMC8958186

[30] Hubli M, Zemp R, Albisser U, Camenzind F, Leonova O, Curt A, et al. Feedback improves compliance of pressure relief activities in wheelchair users with spinal cord injury. Spinal Cord. 2021;59:175–84. 32694751 10.1038/s41393-020-0522-7 PMC7870807

[31] Sundaram SA, Chung CS, Gebrosky B, Brown J, Grindle GG, Deepak N, et al. Participatory action design and engineering of a manual wheelchair virtual coach including in-home and community usage. J Spinal Cord Med. 2023;46:546–59. 35994022 10.1080/10790268.2022.2107352 PMC10274533

[32] Vos-Draper TL, Morrow MMB, Ferguson JE, Mathiowetz VG. Effects of real-time pressure map feedback on confidence in pressure management in wheelchair users with spinal cord injury: pilot intervention study. JMIR Rehabil Assist Technol. 2023;10:e49813. 37824188 10.2196/49813 PMC10603555

[33] Goodwin BM, Olney CM, Ferguson JE, Hansen AH, Eddy B, Goldish G, et al. Visualization of user interactions with a pressure mapping mobile application for wheelchair users at risk for pressure injuries. Assist Technol. 2022;34:444–53. 33395558 10.1080/10400435.2020.1862938 PMC8433259

[34] Moreau-Gaudry A, Chenu O, Dang MV, Bosson JL, Hommel M, Demongeot J, et al. Reduction of prolonged excessive pressure in seated persons with paraplegia using wireless lingual tactile feedback: a randomized controlled trial. IEEE J Transl Eng Health Med. 2018;6:1–11. 10.1109/JTEHM.2018.2842746 PMC6033051 29984117

[35] Yang YS, Chou YC, Hsu JJ, Chang JJ. Effects of audio feedback on sitting behaviors of community-dwelling manual wheelchair users with spinal cord injuries. Assist Technol. 2010;22:79–86. 20698426 10.1080/10400435.2010.483644

[36] Hilgart M, Ritterband L, Baxter K, Alfano A, Ratliff C, Kinzie M, et al. Development and perceived utility and impact of a skin care internet intervention. Internet Interv. 2014;1:149–57.

[37] Houlihan BV, Jette A, Friedman RH, Paasche-Orlow M, Ni P, Wierbicky J, et al. A pilot study of a telehealth intervention for persons with spinal cord dysfunction. Spinal Cord. 2013;51:715–20. 23752260 10.1038/sc.2013.45

[38] Kryger MA, Crytzer TM, Fairman A, Quinby EJ, Karavolis M, Pramana G, et al. The effect of the interactive mobile health and rehabilitation system on health and psychosocial outcomes in spinal cord injury: randomized controlled trial. J Med Internet Res. 2019;21:e14305. 31464189 10.2196/14305 PMC6737885

[39] Schubart J. An e-learning program to prevent pressure ulcers in adults with spinal cord injury: a pre- and post- pilot test among rehabilitation patients following discharge to home. Ostomy Wound Manage. 2012;58:38–49. 23037331

[40] Liu LQ, Deegan R, Dunne H, Knight SL, Allan HT, Gall A. A pilot study for testing feasibility and preliminary influence of early intervention using text messaging for pressure ulcer prevention in individuals with spinal cord injury. J Tissue Viability. 2024;33:666–71. 38964979 10.1016/j.jtv.2024.06.013

[41] Shirai T, Bulandres P, Choi JA, D’Ortenzio D, Moon N, Musselman K, et al. The use of a mobile educational tool on pressure injury education for individuals living with spinal cord injury/disease: a qualitative research study. Disabil Rehabil. 2022;44:468–77. 32493089 10.1080/09638288.2020.1771780

[42] Cogan AM, Blanchard J, Garber SL, Vigen CL, Carlson M, Clark FA. Systematic review of behavioral and educational interventions to prevent pressure ulcers in adults with spinal cord injury. Clin Rehabil. 2017;31:871–80. 27440806 10.1177/0269215516660855

[43] Groah SL, Schladen M, Pineda CG, Hsieh CJ. Prevention of pressure ulcers among people with spinal cord injury: a systematic review. PM R. 2015;7:613–36. 25529614 10.1016/j.pmrj.2014.11.014

[44] Regan MA, Teasell RW, Wolfe DL, Keast D, Mortenson WB, Aubut JAL. A systematic review of therapeutic interventions for pressure ulcers after spinal cord injury. Arch Phys Med Rehabil. 2009;90:213–31. 19236976 10.1016/j.apmr.2008.08.212 PMC3218078

[45] Soegaard K, Sollie M, Beeckman D, Biering-Sørensen F, Ahm-Sørensen J. Interventions, stakeholders, and organisation related to pressure ulcer prevention for individuals with spinal cord injuries in transition from hospital to home - a scoping review. J Tissue Viability. 2023;32:194–205. 36997467 10.1016/j.jtv.2023.02.005

[46] Vos-Draper TL, Morrow MMB. Seating-related pressure injury prevention in spinal cord injury: a review of compensatory technologies to improve in-seat movement behavior. Curr Phys Med Rehabil Rep. 2016;4:320–8. 28603664 10.1007/s40141-016-0140-7 PMC5461958

[47] Baron JS, Sullivan KJ, Swaine JM, Aspinall A, Jaglal S, Presseau J, et al. Self-management interventions for skin care in people with a spinal cord injury: part 1—a systematic review of intervention content and effectiveness. Spinal Cord. 2018;56:823–36. 29802393 10.1038/s41393-018-0138-3 PMC6128818

[48] Liu LQ, Moody J, Traynor M, Dyson S, Gall A. A systematic review of electrical stimulation for pressure ulcer prevention and treatment in people with spinal cord injuries. J Spinal Cord Med. 2014;37:703–18. 24969965 10.1179/2045772314Y.0000000226 PMC4231958

[49] Atkinson RA, Cullum NA. Interventions for pressure ulcers: a summary of evidence for prevention and treatment. Spinal Cord. 2018;56:186–98. 29371701 10.1038/s41393-017-0054-y

[50] Engelen M, van Dulmen S, Vermeulen H, de Laat E, van Gaal B. The content and effectiveness of self-management support interventions for people at risk of pressure ulcers: a systematic review. Int J Nurs Stud. 2021;122:104014. 34274772 10.1016/j.ijnurstu.2021.104014

[51] O’Connor T, Moore ZE, Patton D. Patient and lay carer education for preventing pressure ulceration in at-risk populations. Cochrane Database Syst Rev. 2021;2:CD012006. 33625741 10.1002/14651858.CD012006.pub2 PMC8095034

[52] Fiordelli M, Zanini C, Amann J, Scheel-Sailer A, Brach M, Stucki G, et al. Selecting evidence-based content for inclusion in self-management apps for pressure injuries in individuals with spinal cord injury: participatory design study. JMIR Mhealth Uhealth. 2020;8:e15818. 32432559 10.2196/15818 PMC7270844

[53] Khan A, Phung N. Undergraduate research in assistive technology: design and development of a preventive weight shifting app to reduce the risk of pressure ulcers in wheelchair bound patients with spinal cord injuries (Phase 1). J Med Device. 2016;10:020927.

[54] Chenu O, Vuillerme N, Bucki M, Diot B, Cannard F, Payan Y. TexiCare: an innovative embedded device for pressure ulcer prevention. Preliminary results with a paraplegic volunteer. J Tissue Viability. 2013;22:83–90. 23791763 10.1016/j.jtv.2013.05.002

[55] Oh H, Hye Y, Pontis S. Understanding individuals with spinal cord injury’s self-care practices: a technology probe study to promote pressure relief adherence. Disabil Rehabil Assist Technol. 2024;19:2565–79. 38131605 10.1080/17483107.2023.2293876

[56] Rowland JL, White GW, Wyatt DA. Analysis of an intervention to reduce or prevent secondary conditions for people with spinal cord injuries. J Clin Psychol Med Settings. 2006;13:261–9.

[57] Smit CAJ, Haverkamp GLG, de Groot S, Stolwijk-Swuste JM, Janssen TWJ. Effects of electrical stimulation-induced gluteal versus gluteal and hamstring muscles activation on sitting pressure distribution in persons with a spinal cord injury. Spinal Cord. 2012;50:590–4. 22350033 10.1038/sc.2012.6

[58] Smit CAJ, Legemate KJA, de Koning A, de Groot S, Stolwijk-Swuste JM, Janssen TWJ. Prolonged electrical stimulation-induced gluteal and hamstring muscle activation and sitting pressure in spinal cord injury: effect of duty cycle. J Rehabil Res Dev. 2013;50:1035–46. 24301439 10.1682/JRRD.2012.07.0134

[59] Duffell LD, Donaldson N, de N, Perkins TA, Rushton DN, Hunt KJ, et al. Long‐term intensive electrically stimulated cycling by spinal cord–injured people: effect on muscle properties and their relation to power output. Muscle Nerve. 2008;38:1304–11. 18816613 10.1002/mus.21060

[60] Barton T, Low DA, Thijssen DHJ, Romviel S, Sloots M, Smit CAJ, et al. Twelve-week daily gluteal and hamstring electrical stimulation improves vascular structure and function, limb volume, and sitting pressure in spinal cord injury. Am J Phys Med Rehabil. 2022;101:913–9. 36104843 10.1097/PHM.0000000000001929

[61] Stefanovska A, Vodovnik L, Benko H, Turk R. Treatment of chronic wounds by means of electric and electromagnetic fields. Med Biol Eng Comput. 1993;31:213–20. 8412373 10.1007/BF02458039

[62] Gyawali S, Solis L, Chong SL, Curtis C, Seres P, Kornelsen I, et al. Intermittent electrical stimulation redistributes pressure and promotes tissue oxygenation in loaded muscles of individuals with spinal cord injury. J Appl Physiol. 2011;110:246–55. 20884840 10.1152/japplphysiol.00661.2010

[63] Houghton PE, Campbell KE, Fraser CH, Harris C, Keast DH, Potter PJ, et al. Electrical stimulation therapy increases rate of healing of pressure ulcers in community-dwelling people with spinal cord injury. Arch Phys Med Rehabil. 2010;91:669–78. 20434602 10.1016/j.apmr.2009.12.026

[64] Pellerito JM. The effects of traditional and computer‐aided instruction on promoting independent skin care in adults with paraplegia. Occup Ther Int. 2003;10:1–19. 12830316 10.1002/oti.174

[65] Md Nadzri N, Hamzaid NA, Chung TY. Design and development of a wheelchair seating pressure relief reminder system for pressure ulcer prevention among paraplegics. J Med Eng Technol. 2021;45:574–81. 34184592 10.1080/03091902.2021.1936238

[66] Amann J, Fiordelli M, Scheel-Sailer A, Brach M, Rubinelli S. Opportunities and challenges of a self-management app to support people with spinal cord injury in the prevention of pressure injuries: qualitative study. JMIR Mhealth Uhealth. 2020;8:e22452. 33295876 10.2196/22452 PMC7758166

[67] Amann J, Fiordelli M, Brach M, Bertschy S, Scheel-Sailer A, Rubinelli S. Co-designing a self-management app prototype to support people with spinal cord injury in the prevention of pressure injuries: mixed methods study. JMIR Mhealth Uhealth. 2020;8:e18018. 32673241 10.2196/18018 PMC7380902

[68] Jordan K, Vos-Draper T, Morrow M, Sonenblum S. The usability of two mobile health assistive technologies for wheelchair-related in-seat movement and pressure. J Rehabil Assist Technol Eng. 2023;10:20556683231211808. 38028632 10.1177/20556683231211808 PMC10648002

[69] Motahar T, Ghosh I, Wiese J. Identifying factors that inhibit self-care behavior among individuals with severe spinal cord injury. In: CHI conference on human factors in computing systems. New York, NY, USA: ACM; 2022. pp. 1–16.

[70] Olney CM, Vos-Draper T, Egginton J, Ferguson J, Goldish G, Eddy B, et al. Development of a comprehensive mobile assessment of pressure (CMAP) system for pressure injury prevention for veterans with spinal cord injury. J Spinal Cord Med. 2019;42:685–94. 30702395 10.1080/10790268.2019.1570437 PMC6830274

[71] Prensky, M. Digital natives, digital immigrants part 1. On the Horizon. 2001;9:1–6.

[72] Conti A, Dimonte V, Rizzi A, Clari M, Mozzone S, Garrino L, et al. Barriers and facilitators of education provided during rehabilitation of people with spinal cord injuries: a qualitative description. PLoS ONE. 2020;15:e0240600. 33057362 10.1371/journal.pone.0240600 PMC7561131

