# Supplementary material for: Self-managed digital technologies for pressure injury prevention in individuals with spinal cord injury: a systematic scoping review
Source: Spinal Cord. 2025 Aug 18;63(9):492–8. doi: 10.1038/s41393-025-01113-w (PMC12413319; doi:10.1038/s41393-025-01113-w)
Supplement: Supplementary file 1 — Supplement 1. [file 41393_2025_1113_MOESM1_ESM.docx]

| **Block I** | **Block II** |
| --- | --- |
| \| Spinal cord injury (SH) wo/e \| \| --- \| \| Spinal cord disease (SH) wo/e \| \| Central cord syndrome (SH) wo/e \| \| Cervical spinal cord injury (SH) wo/e \| \| Experimental spinal cord injury(SH) wo/e \| \| Spinal cord compression (SH) wo/e \| \| Spinal cord transection (SH) wo/e \| \| Spinal cord transverse lesion (SH) wo/e \| \| Spinal cord lesion (SH) wo/e \| \| Quadriplegia (SH) wo/e \| \| Spastic paraplegia (SH) wo/e \| \| Paraplegia (SH) wo/e \| \| Spine injury (SH) wo/e \| \| Paralysis (SH) wo/e \| \| Tetraplegi* (ti,ab,kw) \| \| Spastic paraplegi* (ti,ab,kw) \| \| Paraplegi* (ti,ab,kw) \| \| Quadriplegi* (ti,ab,kw) \| \| Paraly* (ti,ab,kw) \| \| (Spin* adj4 contusion*) (ti,ab,kw) \| \| (Spin* adj4 disruption*) (ti,ab,kw) \| \| (Spin* adj4 lesion*) (ti,ab,kw) \| \| (Spin* adj4 trauma*) (ti,ab,kw) \| \| (Spin* adj4 injur*) (ti,ab,kw) \| \| (Spin* adj4 damage*) (ti,ab,kw) \| \| (Spin* adj4 afflict*) (ti,ab,kw) \| \| (Spin* adj4 disease*) (ti,ab,kw) \| \| (Spin* adj4 compression*) (ti,ab,kw) \| \| (Spin* adj4 transsection*) (ti,ab,kw) \| \| (Spin* adj4 transverse lesion*) (ti,ab,kw) \| \| Central cord syndrome* (ti,ab,kw) \| \| Spinal cord disabled (ti,ab,kw) \| \| Spinal cord syndrome (ti,ab,kw) \| \| (Vertebrae adj4 (contusion* or disruption* or lesion* or trauma* or injur* or damage* or afflict*)).ti,ab,kw. \| \|  \| | \| Decubitus (SH) wo/e \| \| --- \| \| Skin injury (SH) wo/e \| \| Skin ulcer (SH) wo/e \| \| Pressure necrosis (SH) wo/e \| \| Soft tissue injury (SH) wo/e \| \| Pressure ulcer* (ti,ab,kw) \| \| Pressure injur* (ti,ab,kw) \| \| Decubit* (ti,ab,kw) \| \| Pressure sore* (ti,ab,kw) \| \| Bedsore* (ti,ab,kw) \| \| Pressure wound* (ti,ab,kw) \| \| Bed sore* (ti,ab,kw) \| \| Pressure lesion* (ti,ab,kw) \| \| Skin ulcer* (ti,ab,kw) \| \| Skin sore* (ti,ab,kw) \| \| Ulcerative wound* (ti,ab,kw) \| \| Ulcerated sore* (ti,ab,kw) \| \| Pressure damage* (ti,ab,kw) \| \| Dermal ulcer* (ti,ab,kw) \| \| Skin injur* (ti,ab,kw) \| \| Pressure necros* (ti,ab,kw) \| \| Shearing wound* (ti,ab,kw) \| \| Friction wound* (ti,ab,kw) \| \| "Moisture-associated skin damage*" (ti,ab,kw) \| \| ("deep tissue" adj2 (injur* or damage* or lesion* or trauma* or laceration* or cut*)).ti,ab,kw \| \| ("soft tissue" adj2 (injur* or damage* or lesion* or trauma* or laceration* or cut*)).ti,ab,kw \| |
