# Supplementary material for: Self-managed digital technologies for pressure injury prevention in individuals with spinal cord injury: a systematic scoping review
Source: Spinal Cord. 2025 Aug 18;63(9):492–8. doi: 10.1038/s41393-025-01113-w (PMC12413319; doi:10.1038/s41393-025-01113-w)
Supplement: Supplementary file 2 — Supplement 2. [file 41393_2025_1113_MOESM2_ESM.docx]

| Author | Country of Origin | Sample  Type | Sample Characteristics | Injury Characteristics | Study Setting | Study Design | Type of Equipment | Study Outcome(s) | Other |
| --- | --- | --- | --- | --- | --- | --- | --- | --- | --- |
| Goodwin et al. (2022) | USA | 6 | Men = 5 (83.3%) Women = 1 (16.7%) Mean age (age range) = 55.3 (39-68) | *Total*  T11 (n =2, 33.3%) T10 (n =1, 16.7%) C5-C6 (n =1, 16.7%) T12-L1 (n =2, 33.3%)  *Individual*  P1 = T11 P2 = T10  P3 = T12-L1  P4 = T12-L1 P5 = T11 P6 = C5-C6 | Cohort A = NA Cohort B = Community setting | Observational study | The AW-Shift© system is a mobile pressure mapping tool comprising a 22×22-inch pressure mat embedded in a custom seat cover, a mobile app (iOS/Android compatible), and a battery-powered Raspberry Pi minicomputer. | Global pressure  Acknowledged global pressure  Local pressure  Acknowledged local pressure  Weight shift reminders ignored  Weight shift reminders snoozed  Weight shift reminders completed |  |
| Hilgart et al. (2014) | USA | 7 | Men = 2 (28.6%) Women = 5 (71.4%) Mean age (age range) = 36.14 SD= 10.09 (NA) | Tetraplegia = 5 (71.4%) Paraplegia = 2 (28.6%) Time since injury = 10.43, SD = 9.64 | Community setting | Pilot study within a larger RCT. | iSHIFTup. a skin care internet containing sixteen modules (aging and skin care, caregivers, employment, equipment and environment, healing, heterotopic ossification, newly injured, nutrition, pressure relief, problem solving, relapse prevention, skin checks, spasticity, tracking changes, transfers, treatment). | Program usage  Internet evaluation and utility questionaire (IEUQ)  Internet impact and effectiveness questionnaire (IIEQ) for iSHIFTup |  |
| Houlihan et al. (2013) | USA | 142 | 142 randomized (SCI = 106, MS = 36).  *NO SCI SPECIFIC DATA (TAKEN FROM 142 TOTAL)*  Intervention group, men = 50 (70.4%)  Intervention group, women = 21 (29.6%)  Control group, men = 37 (52.1%)  Control group, women = 34 (47.9%)  Total group, men = 87 (61.3%)  Total group, women = 55 (38.7%)  Intervention group, mean age (range) = 48.6 SD = 12.5  Control group, mean age (range) = 47.8 SD = 14.1  Total group, mean age (range) = 48.2 SD = 13.3 | SCI intervention = 53 (74.6%)  SCI control = 53 (74.6%)  SCI total = 106 (74.6%)  C1-C8, intervention = 23 (46.0%)  C1-C8, control = 28 (52.8%)  C1-C8, total = 51 (49.5%)  T1-T12, intervention = 25 (50.0%)  T1-T12, control = 21 (39.6%)  T1-T12, total = 46 (44.7%)  L1-L2, intervention = 2 (4.0%)  L1-L2, control = 4 (7.54%)  L1-L2, total = 6 (5.8%)  Incomplete paraplegia, intervention = 18 (37.5%)  Incomplete paraplegia, control = 12 (23.1%)  Incomplete paraplegia, total = 30 (30.0%)  Complete paraplegia, intervention = 9 (18.8%)  Complete paraplegia, control = 15 (28.8%)  Complete paraplegia, total = 24 (24.0%)  Incomplete tetraplegia, intervention = 11 (22.9%)  Incomplete tetraplegia, control = 17 (32.7%)  Incomplete tetraplegia, total = 28 (28.0%)  Complete tetraplegia, intervention = 10 (20.8%)  Complete tetraplegia, control = 8 (15.4%)  Complete tetraplegia, total = 18 (18.0%)  *SCI SPECIFIC*  Age of primary diagnosis onset, mean, intervention = 34.1 SD=15.9  Age of primary diagnosis onset, mean, control = 32.3 SD = 15.7  Age of primary diagnosis onset, mean, total = 33.2, SD = 15.8  Years post-injury, mean, intervention = 11.34 SD = 11.1  Years post-injury, mean, control = 12.0 SD = 9.8  Years post-injury, mean, intervention = 11.7 SD = 10.4 | Community setting | Single-blind randomized controlled trial | CareCall is an automated, interactive voice response system that delivers patient education, cognitive-behavioral support, screening and referrals, and notifies a nurse coordinator for non-urgent follow-up. | Pressure ulcer scale for healing (PUSH) tool version 3.0.11 assisted observation of pressure ulcer prevalence.  The Patient Health Questionnaire—9 depression screener  The Cornell Services Index measured self-reported health-care utilization, hospital visit (yes/no) and emergency room visit (yes/no).  Question 5 of the Craig Hospital Inventory of Environmental Factors-Short Form version 3.0.14 measured self-reported health-care utilization. The availability item (never has problem/has problem) measured at four time points (baseline, 2-month, 4- month and 6-month).  Data collected by the IVR system assessed usage of CareCall. |  |
| Hubli et al. (2021) | Switzerland | 9 | Men = 6 (66.6%) Women = 3 (33.3%) Mean age 50.2 (age range) = (35-75) | Subject 1 = Th10, ASIA A, TsI = 14 years Subject 2 = Th1, ASIA A, TsI = 12 years Subject 3 = Th6, ASIA A, TsI = 10 years Subject 4 = C4, ASIA C, TsI = 1 year Subject 5 = Th8, ASIA A, TsI = 9 years Subject 6 = Th8, AISA A, TsI = 21 years Subject 7 = C6, ASIA A, TsI = 20 years Subject 8 = C4, ASIA C, TsI = 4 years Subject 9 = Th6, ASIA A, TsI = 28 years  *Total* Th10 = 1 (11.1%) Th1 = 1 (11.1%) Th6 = 2 (22.2%) C4 = 2 (22.2%) Th8 = 2 (22.2%) C6 = 1 (11.1%)  ASIA A = 7 (77.7%) ASIA C = 2 (22.2%)  TsI mean (range)= 13.2 years (1-28 years) | In- and out-patient clinic | Prospective cross-sectional pre-post pilot study | The Sensomative Wheelchair system features a textile pressure mat under the cushion, a unit for continuous data monitoring and transmission, and a mobile app that sends personalized pressure relief reminders during regular wheelchair use. | Median values of relief duration and relief frequency measured at three stages= baseline, feedback, and follow-up. |  |
| Kryger et al. (2019) | International (USA and Indonesia) | Intervention = 19 Control = 19 Total = 38 | Intervention, men = 13 (68.4%) Intervention, women = 6 (31.6%) Intervention, mean age (age range) = NA (NA)  Control, men = 12 (63.2%) Control, women = 7 (36.8.%) Control, mean age (age range) = NA (NA)  Total, men = 25 (65.8%) Total, women = 13 (34.2%) Total, mean age (age range) = NA (NA) | Intervention, complete injury = 9 (47.4%) Intervention, incomplete injury = 10 (52.6%) Control, complete injury = 12 (63.2%) Control, incomplete injury = 7 (36.8%)  Total, complete injury = 21 (55.3%) Total, incomplete injury = 17 (44.7%)  Intervention, tetraplegia = 8 (42.1%) Intervention, paraplegia = 11 (57.9%) Control, tetraplegia = 9 (47.4%) Control, paraplegia = 10 (52.6%) Total, tetraplegia = 17 (44.7%) Total, paraplegia = 21 (55.3%)  Intervention, TsI = 9.9, SD = 8 Control TsI = 13.5, SD = 11 | Community setting | Randomized controlled trial | The iMHere mHealth app offers self-management modules for medication (reminders, photo uploads, custom descriptions), bowel/bladder care (reminders, symptom reporting), skincare (photo-based monitoring), mood tracking (validated surveys), and clinician messaging. | Health events per person (wounds) pre-vs-post. study |  |
| Liu et al. (2024) | UK | Intervention = 20 Control = 17 Total = 37 | Intervention, men = 18 (90%) Intervention, women = 2 (10%) Intervention, mean age (range) =49.7 (NA, SD = 15.8)  Control, men = 9 (52.9%) Control, women = 8 (47.1%) Control, mean age (range) = 54.3 (NA, SD = 18)  Total, men = 27 (73%) Total, women = 10 (27%) Total, mean age (range) = 51.8 (NA, SD =16.8) | Duration of injury (months) Intervention = 7.83 (3.7) Control = 7.62 (3.6) Overall = 232 (106)  The level of incomplete or completed injury range from the second cervical (C2) to the first lumbar (L1) | Community setting | Pilot randomized controlledtrial | An automated text messaging system providing educational prompts and motivational messages to support pressure ulcer prevention through skin care, pressure relief, and healthy lifestyle habits. | 8-question acceptability and satisfaction questionnaire  Brief Illness Perception Questionnaire (mBIPQ) to measure perceptions of PrU  Number of skin problems |  |
| Moreau-Gaudry et al. (2018) | France | 9 (control group = 5, intervention group = 4) | Age, Intervention Group - Mean age = 47, SD = 14; Control Group – Mean age = 43, SD = 14  Sex, Intervention Group - 4 Men, 0 Women;  Control Group – 4 Men 1 Woman | All subjects had paraplegia, Control group ASIA scores, 4 = A (80%), 1 = B (20%) Intervention group ASIA scores, 3 = A (75%), 1 = D (25%)  Injury Completeness = Not specified  Time since injury = Not specified | Clinical setting | Randomized, controlled, parallel-group trial | The Tongue Display Unit (TDU) is a wireless electrotactile device with a battery-powered palate and a 6×6 stimulation matrix that signals users via tongue pulses. It pairs with a pressure sensor that transmits data to a laptop; in later sessions, it prompts posture shifts every ~60 seconds based on pressure readings. | Risk Index (R) derived from the synthesis of the following measures=  Instantaneous pressure (mmHg) on one sensor  Indicator function of P>=Prisk (Prisk=100mmHg) of one sensor= 1[P>Prisk]  Instantaneous excessive pressure (IEP(t)) (mmHg)  Instantaneous time-cumulated excessive pressure (IC(t)) (mmHg.ms)  Instantaneous risk (IRt) (ms) |  |
| Schubart (2012) | USA | 15 (1 participant withdrew from the study; he used the program, but chose not to complete the post intervention assessment) | Men = 10 (66.7%) Women = 5 (33.3%) Median age = 37 years Age range = 19-58 | Cervical = 8 (53.3%) Thoracic = 5 (33.3%) Lumbar = 2 (13.3%)  Median months since injury = 72 Range of months since injury = 6-360 | Community setting | Pre- and post- pilot test. | Pressure Ulcer Prevention and Management E-Learning Program | Internet Evaluation and Utility Questionnaire, Internet Impact and Effectiveness Questionnaire, and Internet Adherence Questionnaire.  To test knowledge acquired, an instrument based on the Needs Assessment Checklist (NAC) |  |
| Shirai et al. (2022) | Canada | 9 | Men = 5 (55.6%) Women = 4 (44.4%) Age range = 34-81 Mean age = 59.9 years | T4 = 2 (22.2%) T6 = 2 (22.2%) T12 = 2 (22.2%) T1 = 1 (11.1%) T9 = 1 (11.1%) C6 = 1 (11.1%)  Thoracic = 8 (88.9%) Cervical = 1 (11.1%)  Time since injury range = 3-47 years Mean time since injury = 19.4 years | Community setting | Qualitative study. | Pressure Ulcer Target (PUT), a mobile educational app consisting of six modules= “Skin”, “Pressure Ulcers”, “Staging”, “Prevention”, “Pressure Relief”, and “Management” | Participant quotes |  |
| Sundaram et al. (2023) | USA | *Focus group =* 20 (10 SCI) *Five-day assessment* = 5 *Four-week investigation =* 7 | *Focus group =* Men, clinicians = 3 (15%) Women, clinicians = 7 (35%) Men, SCI = 9 (45%) Women, SCI = 1 (5%) Men, total = (60%) Women, total = (40%)  Clinicians’ mean age (age range) = 47.7 (NA, SD = 17.2) SCI mean age (age range) = 42.1 (NA, SD = 13.2)  *Five-day assessment =* Men = 5 (100%) Women = 0 (0%) Mean age (age range) = 41.0 (NA, SD = 10.3)  *Four-week investigation=*  Men = 5 (71.4%) Women = 2 (28.6%) Mean age (age range) = 44.7 (NA, SD = 11.0) | *No injury characteristics reported other than being SCI*  *Focus group =*  Mean length of wheelchair usage (range) = 11.1yrs (5m – 27yrs)  *Five-day assessment =*  Mean length of wheelchair usage (range) = 18.5yrs, SD = 16.2 yrs (4.5yrs – 44yrs)  *Four-week investigation =* Mean length of wheelchair usage (range) = 24.7yrs, SD = 17.0 yrs (6yrs – 47yrs) | Community setting | Participatory action design and engineering (PADE) study. | The Manual Wheelchair Virtual Coach (MW-VC) includes load cell hardware, electronics, a smartphone app, and a cloud database. It monitors center of pressure, body weight, and pressure relief (PR), sending data via Bluetooth to an Android app. The app displays real-time metrics, provides PR guidance, sends alerts if no effective PR occurs within 30 minutes, and uploads daily usage data for feedback collection. | Focus group quotations  Four-week intervention quotations  Center of pressure (CoP) data (Fig 5)  Daily mean and standard deviation of occupancy time (Table 1)  Time in each PR area (Table 1)  Number of times entering each PR area (Table 1)  Number of complete PR of each type (>2 min in the PR area) (Table 1) |  |
| Vos-Draper et al. (2023) | USA | In-clinic visit (total) (n=23) In-home phase (n=16) | *In-clinic visit (total)*  Men = 18 (78.3%) Women = 5 (21.7%) Mean age (age range) = 42.17 (NA), SD = 13.16  *In-home phase* Men = 11 (68.8%) Women = 5 (31.3%) Mean age (age range) = 42.5 (NA), SD = 12.38 | In-clinic visit (total) Cervical SCI = 10 (43.5%) Thoracic SCI = 12 (52.2%) Lumbar SCI = 1 (4.3%) Mean years since onset (range) = 15.74 (NA), SD= 11.77  In-home phase Cervical SCI = 6 (37.5%) Thoracic SCI = 9 (56.3%) Lumbar SCI = 1 (6.3%) Mean years since onset (SD) = 18.13 (11.40) | Clinic, transitioned to community | Pre-post, longitudinal, within-subject, repeated measures design. | The mPAP is a real-time pressure mapping system that wirelessly connects to a BodiTrac 4-way stretch pressure mat, displaying continuous pressure data on a smartphone screen. | 4-item self-efficacy survey.  Mean (SD) and 95% CI values in response to the statements= “I believe I am able to… prevent pressure injury using weight shifts” and “I believe I am able to… move far enough to relieve pressure. |  |
| Yang et al. (2010) | Taiwan | 20 | Men = 18 (90%) Women = 2 (10%) Mean age= 37 (SD = 10.3) | Paraplegia = 11 (55%) Tetraplegia = 9 (45%) SCI ranging from C5 to L2 Mean time since injury = 5.8 years (SD = 5.9/6.0) | Community setting | Quasi-experimental, pre-post design. | The data logger includes a Tattletale TFX-11 microcontroller and six force sensors embedded in a mat—two front, four rear—to monitor weight distribution near the thighs and ischial tuberosities. An integrated audio alarm provides alerts. | Cumulative sitting time per day (min)  Average time of uninterrupted sitting (min)  Frequency of daily transfers from wheelchair  Frequency of push-up in wheelchair (times/day)  Frequency of leaning forward in wheelchair (times/day)  Frequency of side-to-side leaning in wheelchair (times/day)  Total frequency of pressure-relief activities (times/day) |  |
