# Supplementary material for: Self-managed digital technologies for pressure injury prevention in individuals with spinal cord injury: a systematic scoping review
Source: Spinal Cord. 2025 Aug 18;63(9):492–8. doi: 10.1038/s41393-025-01113-w (PMC12413319; doi:10.1038/s41393-025-01113-w)
Supplement: Supplementary file 3 — Supplement 3. [file 41393_2025_1113_MOESM3_ESM.docx]

| Author (year) | Study Intervention | Main Results |
| --- | --- | --- |
| Goodwin et al. (2022) | Two participant cohorts (A and B) received in-person setup and training on a wheelchair-mounted pressure mat system and the AW-Shift© app. Cohort A used their personal phones; Cohort B received study-issued phones with the app pre-installed. Participants customized pressure alert and weight shift reminder settings with guidance from an occupational therapist. Cohort A used the system for 7 days; Cohort B was assigned 4 weeks, but only the first 7 days were analyzed. The system logged weight shift responses (completed, snoozed, ignored), pressure alerts (acknowledged or not), and app interactions. | Participants 1 and 4 set hourly weight shift reminders, while Participants 2 and 5 chose intervals over 12 hours. Only Participant 6 consistently completed prompts; others showed low adherence during the week.  SEE TABLE 3 SEE FIGURES 3 AND 5 |
| Hilgart et al. (2014) | Over four weeks, iSHIFTup tracked daily skin care habits (sitting time, pressure reliefs, skin checks). It began with three Core units on PrU risk, prevention, and behavior strategies. Weekly follow-ups assessed adherence and guided personalized modules. The program used interactive content, prompts, and goal-setting to support engagement. | Participants reported that iSHIFTup improved their skin care routines and PrU prevention knowledge. Most (86%) rated it highly effective for supporting skin care, and 71% found it personally effective. The majority felt confident in meeting goals (86%) and valued the diary feature (71%).  Regarding behaviors, 71% said the program helped with regular pressure relief, increased frequency, and skin checks. All users noted improvements in pressure relief duration, identification of at-risk areas, and skin assessment skills. Most (86%) found it beneficial for overall skin health and timely response to skin issues (71%). All participants believed it supported PrU prevention and would recommend it to others.  SEE TABLE 2  SEE FIGURE 7 |
| Houlihan et al. (2013) | Before randomization, participants completed a training call to ensure CareCall usability. The intervention group received weekly automated calls for six months, with optional call-in access. Calls featured modules on skin care, depression, and healthcare use, with messages from peers and professionals. Content was tailored via branching logic.  The control group received usual care and a CareCall resource book with expert information and local resources. Outcomes were assessed at baseline and six months via home visits, with healthcare use data collected at two and four months. | Overall, CareCall did not significantly reduce pressure ulcer incidence at six months after adjusting for baseline variables. However, women in the intervention group showed a significant reduction, with no ulcers reported at follow-up (P = 0.04; P < 0.0001). No such effect was seen in men (P > 0.05). Although men were 4.5 times more likely to report turning in bed every 2–4 hours (P = 0.008), this did not lower ulcer rates. Other behaviors, like seated pressure relief, showed no gender differences. Results suggest CareCall may be less effective for men in ulcer prevention.  SEE FIGURE 2 |
| Hubli et al. (2021) | The study evaluated the impact of feedback on pressure relief over three weeks. Week 1 (baseline) involved passive data collection via a wheelchair-mounted pressure mat. In Week 2 (feedback phase), participants used a mobile app guided by an OT to set personalized pressure relief parameters and practiced relief techniques. Real-time alerts and reminders supported adherence. In Week 3 (follow-up), feedback was removed to assess retention, while sitting behavior continued to be recorded. | Relief Frequency:  All participants increased their pressure relief frequency during the feedback phase compared to baseline and follow-up. A significant difference was found across the three weeks (χ²(2) = 13.556, p = 0.001). Post hoc tests showed a moderate effect from baseline (median 11%) to feedback (82%; r = −0.628), and from feedback to follow-up (20%; r = −0.628). No significant difference emerged between baseline and follow-up (r = −0.154), suggesting the effect was not maintained without feedback.  Relief Duration:  Two participants lacked sufficient baseline data for median duration analysis. Those below the recommended durations initially showed improvements with feedback, while three participants already met targets in week one. One participant (Subject 4) relied solely on backward tilt using a powered wheelchair. Relief duration varied significantly across weeks (χ²(2) = 6.500, p = 0.039), with moderate effects across pairwise comparisons (r range: −0.646 to −0.517), indicating gains during feedback were not sustained afterward.  SEE TABLES 2 AND 3  SEE FIGURES 2 AND 3 |
| Kryger et al. (2019) | After baseline data collection, participants were randomized. The control group received standard outpatient care, including periodic visits with an SCI-trained physician. The intervention group received the iMHere app on a Samsung Galaxy S5 plus standard care. A 30-minute training covered app use, reminders, and data entry. Participants used only modules relevant to their routine, with reminders integrated into their self-management plan. If a pressure injury was found, they uploaded a photo and its location via the app. A physical therapist monitored submissions via a web portal and provided electronic feedback. Health outcomes were evaluated through retrospective chart reviews spanning the 9 months before and during the study, and all participants completed phone interviews at baseline and at three-month intervals. | Wounds pre-study, intervention: 0.3 Wounds post-study, intervention: 0.1  Wounds pre-study, control: 0.2 Wounds post-study, control: 0.2  No significant changes were observed in any primary outcome measures aside from UTIs in both the intervention and control groups.  SEE FIGURE 3 |
| Liu et al. (2024) | Participants were randomly assigned to receive either standard care or a six-month intervention with twice-weekly text reminders after hospital discharge. Messages offered education and motivation for pressure ulcer (PrU) prevention, addressing skincare, pressure relief, and healthy habits. The first four weeks featured themed content (e.g., skincare, early detection, hydration/nutrition, equipment use), followed by ongoing reminders on posture and skin checks. A welcome message introduced the program. Outcomes—including prevention behaviors and PrU incidence—were assessed at baseline and six months. | At six months, the text message group showed improved adherence to pressure ulcer prevention. Pressure relief frequency increased from 71.7% to 86.7% (p = 0.02), and skin inspections rose from 4.75 to 6.75 days per week (p = 0.04). Participants reported fewer concerns (p = 0.04) and barriers (p = 0.01) to performing reliefs. Perceived seriousness of PrUs increased slightly (67.1% to 69.6%, p = 0.05), and nearly all participants reported greater confidence in prevention, with one strongly agreeing.  SEE TABLES 2 AND 3 |
| Moreau-Gaudry et al. (2018) | Participants were randomized to either a control group (no feedback) or an intervention group using the Tongue Display Unit (TDU). Each completed two one-hour sessions (S1 and S2), spaced one week apart, while seated on a pressure sensor mat. In both S1 and S2 for controls, no feedback was given. In S2 for the intervention group, the TDU provided directional tongue cues every 60 seconds to prompt posture adjustments. Signals stopped upon achieving target posture or after 10 seconds. Buttock pressure was monitored continuously during both sessions while participants watched a movie. | The intervention group showed significantly greater improvements in proper weight shifting than controls and a trend toward reduced prolonged pressure. While the pressure sensor was deemed suitable for daily use, the TDU was not considered practical for regular application. |
| Schubart (2012) | Participants began with a nurse-administered Needs Assessment Checklist (NAC), then received a self-paced, two-week e-learning program on pressure ulcer prevention via email. Post-intervention telephone questionnaires followed completion of the Learning section. At follow-up, the Internet Evaluation and Utility Questionnaire was administered. A researcher remained available for technical support and accessibility assistance throughout the study. | Eleven participants reported improved knowledge of pressure ulcers after completing the program. In terms of confidence, four felt "mostly" and ten "very" confident in prevention and detection. Adherence ratings varied: two "slightly," ten "somewhat," and two "mostly" or "very" followed recommendations.  Skin Care Knowledge and Practice Questionnaire scores improved from a median of 96 to 107 and a mean of 92 to 106 (range: 70–100 to 97–114; max score: 120). Significant gains were seen in skin check and PrU prevention knowledge (p < 0.05), though other changes were not statistically significant.  SEE TABLE 5 |
| Shirai et al. (2022) | The Pressure Ulcer Target (PUT) app was evaluated over two weeks, followed by semi-structured interviews with nine participants (seven in person, two by phone). During interviews, participants used the app on an iPad to contextualize responses and align feedback with usage. Interviewers recorded non-verbal cues and navigation behaviors through field notes, supported by a second researcher to enhance observational accuracy. | Participants found PUT helpful for reinforcing prevention strategies. One noted it encouraged thinking about “measures [they] should take to prevent pressure sores.” Modules on prevention, staging, and tissue healing enhanced knowledge and self-management. One participant shared that “it [does] not [seem] that hard to prevent [PIs] after reading this,” while others valued learning about “the stages of the skin breaking down” and that “even after a year [it] is not [as] strong as the normal tissue.” Additionally, PUT served as a reminder tool, with one stating it “made [her] much more aware of getting the weight off [her] backside and doing pressure relief [strategies].” |
| Sundaram et al. (2023) | The MW-VC was evaluated in three phases:  Focus Groups: Clinicians and manual wheelchair users (MWUs) discussed usability, functionality, training, and improvements to the MW-VC prototype. MWUs were invited to join future trials.  Five-Day In-Home Study: Participants used the customized MW-VC system at home and in the community for five days. Sitting behavior was recorded, and post-trial feedback guided refinements.  Four-Week In-Home Study: The updated system was tested over four weeks to assess long-term usability, sitting behavior, pressure relief patterns, and system reliability. | Focus Groups:  Participants discussed the MW-VC’s potential to support more frequent pressure relief, especially for new MWUs during rehab and community integration. Some believed users might outgrow the need for reminders as habits formed. One participant suggested that alarms after missed reliefs could reinforce behavior change.  Five-Day In-Home Study:  No evaluation data reported.  Four-Week In-Home Study:  Participants reported mixed experiences with reminders—some received prompts inaccurately, while others received none. Despite these issues, all completing participants saw promise in the device but emphasized the need for improvements.  SEE TABLE 1  SEE FIGURE 5 |
| Vos-Draper et al. (2023) | In-Clinic Visit:  Participants completed a baseline self-efficacy (SE) survey before receiving tailored instruction on weight shift techniques for pressure injury (PI) prevention. Under expert supervision, they practiced full and partial leans (including 45–55° full tilts and 30° partial tilts for power tilt users). After training, they completed a second SE survey, then used real-time interface pressure mapping (IPM) to visualize pressure shifts while practicing maneuvers. A third SE assessment followed.  In-Home Use of mPMAP:  Participants used the mPMAP system and an iPhone in a 30-day ABAB trial (alternating weekly between use and non-use, starting with use). Before the trial, they demonstrated independent system operation. They recorded daily activity and usability feedback, and completed five SE surveys throughout the trial. | Confidence in the effectiveness of weight shifts for PI prevention significantly increased after standard education (mean rose from 85.2 to 90.2; p = .02, r = –0.503). Confidence further improved with IPM feedback in the clinic (mean 94.3) and remained high during the one-month home trial (mean 93.8), though these additional gains were not statistically significant and had a small effect size.  SEE TABLES 2 AND 4 |
| Yang et al. (2010) | Participants reported demographics and wheelchair sitting time before a data logger and FSR mat were installed on their wheelchairs (secured under the cushion if used). Over two separate weeks, sitting behavior was monitored. In Week 1, data were collected without feedback as participants followed their normal routines. In Week 2, an audio alarm was activated, prompting participants to perform pressure-relief activities (push-ups or side leans) when signaled. Data were archived at the end of each week to evaluate changes in pressure-relieving behavior. | Sitting Time:  Average cumulative sitting time remained consistent with and without the audio alarm (553 vs. 552 minutes), aligning with the self-reported average of 9.6 hours/day. No significant differences in sitting time were observed across the two weeks (p = .65), and sitting/transfer behaviors remained stable. Age and injury duration were not significantly correlated with sitting variables (p > .05).  Pressure Relief Activities:  Audio alarms significantly increased total pressure-relief activities—from 14.7 to 17.6 per day (p = .01). Significant gains were seen for push-ups (p = .03) and side leans (p = .04), but not forward leans (p = .49). Uninterrupted sitting time also decreased significantly, from 97 to 84 minutes (p = .02), during the feedback week.  SEE TABLE 2 |
