# Supplementary material for: Self-managed digital technologies for pressure injury prevention in individuals with spinal cord injury: a systematic scoping review
Source: Spinal Cord. 2025 Aug 18;63(9):492–8. doi: 10.1038/s41393-025-01113-w (PMC12413319; doi:10.1038/s41393-025-01113-w)
Supplement: Supplementary file 4 — Supplement 4. [file 41393_2025_1113_MOESM4_ESM.docx]

Records inaccessible at the FT screening level consisted of:

1. #19955 - Amosu 1998
   Challenges of nursing paraplegics and quadriplegics with pressure sores at National Orthopaedic Hospital in Nigeria.
   Amosu OO
   West African Journal of Nursing May 1998;9(1):46-50
   West African College of Nursing 1998 May
   Ref ID: 107215364
2. #14505 - Anisha 2019
   Prevention of bedsore in physically disabled patients
   Anisha, M.; Vishnuvarthanan, G.; Janani, K.; Golda, S.M.; Poornima Devi, M.
   J. Adv. Res. Dyn. Control. Syst. 2019;11(6 Special Issue):193-199
   Institute of Advanced Scientific Research, Inc. 2019
3. #5855 - Anonymous 1974
   The Steeper Co-Ro Bed
   Anonymous.
   Rehabilitation / 1974;89():27
   1974 /
4. #5538 - Chalker 1983
   Healing of decubitus ulcers of patients in neuro-kinesthetic program with the Electro-Acuscope 80
   Chalker M.
   Critical care update / 1983;10(3):50-52
   United States 1983 /
   Ref ID: 6601565 [https://www.ncbi.nlm.nih.gov/pubmed/?term=6601565]
5. #20371 - Engel 2008
   Developing and implementing a transdisciplinary educational model for the prevention of pressure ulcers.
   Engel KL; Waring WP III
   SCI Nursing Spring 2008;25(1):31-39
   Jackson Heights, New York American Association of the Spinal Cord Injury Nurses 2008 Spring
   Ref ID: 105813851
6. #20388 - Fine 1996
   Skin care.
   Fine CK
   Topics in Spinal Cord Injury Rehabilitation Summer 1996;2(1):v-56
   Brainerd, Minnesota KnowledgeWorks Global, Ltd 1996 Summer
   Ref ID: 107338904
7. #21276 - Kestsumpun 1999
   Empowerment: a clinical approach for positive patient outcomes in Thailand.
   Kestsumpun Y
   World Council of Enterostomal Therapists Journal Apr-Jun 1999;19(2):10-12
   Cambridge Publishing 1999 Apr-Jun
   Ref ID: 107199343
8. #20697 - Preston 2003
   Scales of pressure: an assessment toolbox for preventing pressure sores in individuals with SCI.
   Preston MA
   Rehab Management: The Interdisciplinary Journal of Rehabilitation Jun 2003;16(5):34-59
   Overland Park, Kansas MEDQOR 2003 Jun
   Ref ID: 106703705
9. #21419 - Rappl 2006
   Dealing with bedrest.
   Rappl LM
   Acute Care Perspectives Summer 2006;15(2):8-9
   Pittsburgh, Pennsylvania Acute Care Section - APTA, Inc. 2006 Summer
   Ref ID: 106289470
10. #3626 - UnluSakaci 2010
    Spinal cord injury in a patient with ankylosing spondylitis: Complications with atypical course: Case report
    Unlu Sakaci I.; Ersoz M.; Tunc H.; Firat M.; Akkus S.
    Journal of Rheumatology and Medical Rehabilitation / 2010;21(1):17-20
    Turkey Hacettepe Universitesi Tip Fakultesi (Ankara 06100, Turkey) 2010 /
11. #20817
    Keeping current: electrical stimulation can augment standard pressure ulcer management.
    Teichs T; Purfield J
    Advance for Physical Therapy & Rehab Medicine ;21(16):28-39
    Pennsylvania Merion Publications
    Ref ID: 104965128
